# Design Principles of Hybrid Nanomaterials for Radiotherapy Enhanced by Photodynamic Therapy

**DOI:** 10.3390/ijms23158736

**Published:** 2022-08-05

**Authors:** Valeria Secchi, Angelo Monguzzi, Irene Villa

**Affiliations:** 1 Department of Materials Science, University of Milano-Bicocca, Via R. Cozzi 55, 20125 Milan, Italy; 2 NANOMIB, Center for Biomedical Nanomedicine, University of Milano-Bicocca, P.zza Ateneo Nuovo 1, 20126 Milan, Italy; 3 Institute of Physics of the Czech Academy of Sciences, FZU, Cukrovarnická 10/112, 16200 Prague, Czech Republic

**Keywords:** radiotherapy, photodynamic therapy, nanoscintillators, singlet oxygen, nanoparticles, energy sharing

## Abstract

Radiation (RT) remains the most frequently used treatment against cancer. The main limitation of RT is its lack of specificity for cancer tissues and the limited maximum radiation dose that can be safely delivered without damaging the surrounding healthy tissues. A step forward in the development of better RT is achieved by coupling it with other treatments, such as photodynamic therapy (PDT). PDT is an anti-cancer therapy that relies on the light activation of non-toxic molecules—called photosensitizers—to generate ROS such as singlet oxygen. By conjugating photosensitizers to dense nanoscintillators in hybrid architectures, the PDT could be activated during RT, leading to cell death through an additional pathway with respect to the one activated by RT alone. Therefore, combining RT and PDT can lead to a synergistic enhancement of the overall efficacy of RT. However, the involvement of hybrids in combination with ionizing radiation is not trivial: the comprehension of the relationship among RT, scintillation emission of the nanoscintillator, and therapeutic effects of the locally excited photosensitizers is desirable to optimize the design of the hybrid nanoparticles for improved effects in radio-oncology. Here, we discuss the working principles of the PDT-activated RT methods, pointing out the guidelines for the development of effective coadjutants to be tested in clinics.

## 1. Introduction

Cancer is one of most threatening diseases to the human population. To eliminate it, in recent decades radiotherapy (RT) has been progressively developed and refined, becoming one of the most diffused and affordable treatments of oncological pathologies in clinics [1,2]. Ionizing radiation with photon energies from keV to MeV is able to penetrate into the human tissues and damage cancer cells, limiting the tumor growth or killing it. RT outcomes are closely entangled with the chance to monitor tumor regression. Thus, RT can be coupled to advanced biological imaging methodologies—such as positron emission tomography, computed tomography, and magnetic resonance imaging. The combination of RT with modern imaging techniques allows the optimization of dose planning and delivery of RT according to the functional imaging information, resulting in a superior outcome and reduction in toxicity with better quality of life for the cancer patients. Nowadays, RT represents one of the leading cancer treatment approaches, which addresses the needs of more than 70% of patients, both as main treatments or as a coadjuvant for chemotherapy or surgery [3]. However, the current RT treatments exploit high delivered doses, which are far from safe for the human body as they can generate radiation-induced toxicity in the healthy tissue that lies close to the cancer [4]. Hence, current research is still focused on finding a striking trade-off between inhibiting tumor growth and reducing side effects to normal tissue through fine control of the dose of radiation administered to patients.

Photodynamic therapy (PDT) has been proposed as an alternative to RT due to its high selectivity and minimal invasiveness [5]. The PDT is based on photosensitizer moieties selectively activated by light in the presence of oxygen, which produce highly cytotoxic reactive oxygen species (ROS) that can kill the cancer cells. PDT has been utilized in the clinic for treatments of different cancer types; however, despite the excellent results obtained, its clinical use is actually limited by the shallow tissue penetration of light, especially in the visible spectrum window where most of the best photosensitizers can absorb the incoming photons [6,7]. Therefore, starting from the early 2000s, research in the PDT field proposed to use highly energetic radiation such as X-rays to activate the photosensitizers instead of low-energy photons [8,9,10]. The high tissue penetrability of X-rays would in principle activate the PDT agents even in deep tissues [11], to obtain an improved therapeutic outcome and, simultaneously, a reduced radiation damage to normal tissue for the treatment of deep-seated cancer [12].

A further step to overcome the PDT drawbacks was made in 2006, with the introduction of the concept of energy transducers to transform the energy deposited by X-rays into optical luminescence that can activate the PDT moieties, thus achieving an enhancement of the RT effect by X-ray-activated PDT (X-PDT) [13]. The energy transducers are usually luminescent nanoparticles, i.e., nanoscintillators, that can interact with the ionizing radiation, achieving a photon down-conversion from the X-ray spectral range into the visible range [14]. The presence of these nanoscintillators allows (i) the promotion of energy deposition localized in the tissue of interest and (ii) the activation of the PDT agents in deep tissues [15,16,17]. To date, diverse classes of inorganic dense nanoscintillators have been combined with organic photosensitizers for X-PDT applications [18], and they have been investigated both in vitro and in vivo [10,19,20,21,22]. The obtained results demonstrate unambiguously that the X-PDT approach results in a synergistic therapeutic effect of RT and PDT, with the final curative efficiency being more than the sum of the results produced individually by the two treatments [23,24,25,26,27]. Nevertheless, besides these encouraging results, a complete understanding of the energy-sharing mechanisms that occurs in the scintillation process between the X-PDT species and the biological environment is still lacking. Consequently, the general guidelines for the design of optimized X-PDT systems to be tested in a clinical environment are eagerly sought out.

The aim of this work is to highlight the general key parameters to optimize the performance of any multicomponent nanomaterial proposed for X-PDT applications. Indeed, appropriate design of the system, in terms of its composition and concentration of the constituents, as well as type of linkage strategy among the components can affect the interaction with the ionizing radiation, the activation of the photosensitizers, and the production of ROS, and thus the overall therapeutic efficacy of the X-PDT agent. Diverse potential archetypal architectures of multicomponent hybrid nanomaterials for X-PDT are discussed, pointing out a possible guideline for the realization of efficient materials that are exploitable in future clinical trials.

## 2. Principles of Radiotherapy

**Figure 1:**
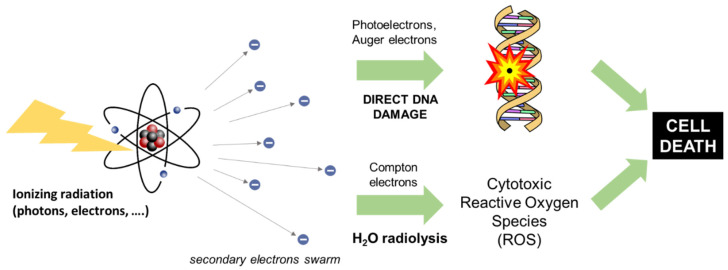
Sketch of the radiotherapy (RT) mechanism in living tissues. Upon interaction on the incident ionizing radiation with matter, secondary charges can induce cellular death by (i) directly damaging their DNA or (ii) by enhancing the water radiolysis reaction that produces highly cytotoxic reactive oxygen species (ROS).

RT is the principal tool for the cure of many malignancies, such as breast carcinoma, lung carcinoma, melanoma, gastrointestinal cancers, head and neck cancers, gynecological cancers, hematologic malignancies, prostate or cervical tumors, central nervous system neoplasms, and thyroid carcinomas [3]. As illustrated in Figure 1, RT exploits the damage produced by ionizing beams—X-rays, γ-rays, or radiation emitted by radionuclides—to stop the rapid proliferation of cancer cells and/or to treat cancer symptoms as palliative treatment for an effective reduction in pain [3,28]. When a high-energy photon beam interacts with the biological medium, three main physical phenomena occur: the photoelectric effect, Compton scattering, and pair production. The relative probabilities of each interaction are simple functions of the energy of the incident photon and the atomic number (Z) of the target atom. Under clinical RT conditions, the Compton and photoelectric effects are dominant, and the deposition of their energy occurs through a cascade of secondary photoelectrons and Auger electrons [29]. As depicted in Figure 1, the interaction of high-energy radiation within tissues can kill the tumor cells directly thanks to the damage of the DNA molecular structure by generated secondary electrons. This damage blocks cellular division and proliferation, thus activating the metabolic patterns that lead to programmed cell death. On the other side, cancer cell destruction can arise indirectly thanks to the radiation-induced formation of cytotoxic ROS within the cellular aqueous environment [30,31,32]. In a cellular environment, ionizing radiation induced water radiolysis with the consequent formation of several species, mainly: e_aq_- (hydrated electrons), HO^•^ (hydroxyl radicals), H^•^ (hydrogen radicals), H_2_ (radiolytic hydrogen), H_2_O_2_ (hydrogen peroxide), and HO_2_^•^ (hydroperoxyl radical). After further reaction mechanisms, these products can create additional ROS, such as ${O_{2}}^{•-}$) (superoxide), organic radicals (R^•^), hydroperoxides (ROOH), and singlet oxygen (^1^$O_{2}$*). This additional ROS production perturbs the ROS balance established in cells under physiological conditions by the antioxidant system to maintain cellular homeostasis. Therefore, the radiation-induced oxidative stress condition again leads to DNA damage and consequently to cell death by several mechanisms such as necrosis, apoptosis, autophagy, mutation, and senescence [33,34]. RT can be coupled to other oncological therapies including surgery, chemotherapy, or immunotherapy [35,36]. Besides high-energy photons, other types of ionizing radiation such as electron and hadron (e.g., neutrons, protons, and heavy ions) beams can be exploited under specific therapeutic conditions [37,38]. In this work, we focus on RT based on photons.

According to diverse factors involving the type and size of cancer, the location in the body, the volume in relation to the normal tissue and healthy organs, and the medical history of the patient, the RT healing dose can be delivered to the tumors by external or internal methodologies [39]. The internal RT methodology requires the use of solid or liquid radioactive sources inserted inside the fluids of the human body (targeted radionuclide therapy) [40,41] or in proximity to the tumoral lesions into a body cavity/lumen/interstitial location using a probe coupled to radioactive elements such as ^198^Au, ^60^Co, ^137^Cs, ^32^Ph, and more recently, ^132^Pd and^125^I (brachytherapy). The delivered dose, in this case, depends on the radionuclide activity and on the number of radionuclides successfully accumulated in the tissues of interest [42,43]. Conversely, the external RT refers to all the procedures involving the use of high-energy external photon beam (both X-rays and γ-rays). In this work, we focus on RT applied by an external source. For the treatment of tumors > 2 cm in depth such as in breast cancers, controlled photons are generated by linear accelerators working within the energy range of 4–25 MV [44]. On the other side, low-energy X-ray beams up to 200 kV can deliver the maximum dose very close to the human tissue surface; thus, low-energy beams are dedicated to melanoma, basal cell carcinoma, squamous cell carcinoma, and keloid treatments located at a depth < 5 mm [45]. The current operative regimes for external RT are based on the linear quadratic (LQ) mathematical model to describe the damage to the replication ability of the cancer cells caused by the hit of the ionizing radiation [46]. In this model, the surviving fraction of cells S(D) after a dose of radiation D is given by

S(D) = exp (−αD − βD^2^) (1)

where α is the coefficient of non-repairable damage and β is the coefficient of repairable damage. Considering that the proliferation rate of the normal cells is slower than that of cancer cells (i.e., cancer cells display low values of β), a significant improvement in the efficacy of RT has been accomplished by the establishment of the fractionated regime. In this case, the protocol dose is given in a succession of small doses: after each fraction, only normal healthy cells can repair some of the radiation damage. The optimization of the fractionated regime is based on the critical trade-off among the dose/fraction, time interval between the fractions, and the total RT dose, though, in established RT protocols, patients are typically treated through a fractionated regime to reach the required therapeutic dose delivery in a time range spanning from 4 to 8 weeks according to the disease characteristics [47]. 

The lack of selectively is the major drawback of the RT approach. In this regard, the progress in external RT technology has allowed the establishment of different methodologies for more precise dose delivery while saving the normal organ structures from undesirable irradiation. Advanced beam managing techniques allow to act accurately on deep parts of the body. For example, γ-ray beams are exploited for the treatment of lesions and tumors located in the brain, where multiple photon beams (Gamma Knife) are precisely focused to deliver the therapeutic dose with extremely high accuracy in space [48,49]. Three-dimensional conformal radiotherapy (3DCRT) allows optimal beam placement and shielding towards a more localized dose delivery in the malignancy. Similarly, intensity-modulated radiation therapy (IMRT) is based on the combinations of multiple intensity-modulated fields coming from different beam directions, in combination with imaging techniques, to produce a customized radiation dose that optimizes the localized dose delivery—conforming to the three-dimensional shape of the tumor—while minimizing the dose to adjacent normal tissues. Image-guided radiotherapy (IGRT) is an advanced technology to control the progress of the RT on the tumor and on the adjacent critical organs. IGRT counts on pre-radiotherapy imaging, such as CT scans, which allows the application of dose-delivery corrections/patient-position modifications according to the tumor change in volume [50,51]. 

## 3. Principles of Photodynamic Therapy

**Figure 2:**
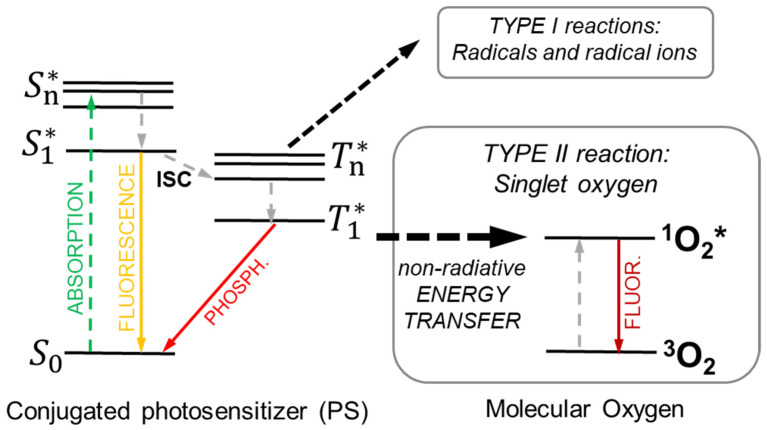
Outline of the photophysical process involved in the sensitization of singlet oxygen (^1^$O_{2}$*) production in photodynamic therapy. Dashed lines marks non-radiative transitions. Upon absorption of a photon, the selected conjugated photosensitizer molecule is promoted in its excited singlet state ($S_{1}^{*}$). Subsequently, the absorbed energy is partially transferred to the PS triplet $T_{1}^{*}$ by ultrafast intersystem crossing (ISC), from which a non-radiative energy transfer promotes the triplet ground state of molecular oxygen ^3^$O_{2}$ dispersed in the environment to its excited singlet state.

Photodynamic therapy (PDT) is a clinically approved cancer therapy based on photochemical reactions. It relies on the interplay between light-triggered molecules so-called photosensitizers, photons, and molecular oxygen. The reaction of a light-activated photosensitizer with the latter created radicals or reactive oxygen species (ROS) [52], which can directly damage cells and/or the vasculature, as well induce inflammatory and immune responses. The PDT is therefore a two-step procedure, which starts with photosensitizers administration followed by a locally directed light exposure, with the aim of confined tumor destruction. The photophysics involved in the creation of ROS is summarized in Figure 2. The absorption of a photon promotes the photosensitizers to an excited singlet state $S_{n}^{*}$, from which the system thermalizes to it first excited singlet $S_{1}^{*}$. Here, the molecule can recombine to its ground state by producing fluorescence, a phenomenon widely exploited in diagnostics and imaging [53] or populate its lowest triplet state $T_{1}^{*}\;$ by ultrafast intersystem crossing (ISC). In this $T_{1}^{*}\;$ state, the photosensitizer can transfer its energy by phosphorescence or by colliding with other molecules to create chemically reactive species via two types of reactions [53,54]. The so-called Type I reaction occurs when the $T_{1}^{*}\;$ state reacts with organic substrates or solvents, thus transferring an electron or a proton to form radical anion or cation species, respectively. For example, the photosensitizers typically react with an electron-donating substrate to form reduced photosensitizers PS^(−)^ that subsequently react with molecular oxygen to form superoxide anion radicals (${O_{2}}^{•-}$). In the Type II reaction, the $T_{1}^{*}\;$ reacts directly with a ground state oxygen ^3^$O_{2}$ molecule by non-radiative Dexter energy transfer (ET, Section 4.1) to promote the molecular oxygen to its excited singlet state (^1^$O_{2}$*), which is another highly cytotoxic ROS [54,55].

The effects of PDT can be differentiated in three main areas. The first one is the direct consequence of the presence of high amounts of ROS, which result in cytotoxicity as both products superoxide and singlet oxygen can directly react with and damage biomolecules such as lipids, proteins, and nucleic acids [56]. The superoxide anions formed in Type I reactions are not particularly damaging in biological systems directly but can be part of a reaction that produces hydrogen peroxide [57]. Nevertheless, most photosensitizers act through Type II reactions in which singlet oxygen is the main molecule causing oxidative cellular damage, perturbing the cellular metabolism, thus inducing cell death by apoptosis, necrosis, and autophagy, depending on the photosensitizer’s localization in a specific cellular district. Since singlet oxygen is highly reactive, its lifetime is in the order of 40 ns and has a maximum action radius of about 20 nm [58]. This short action radius means a strong localization of the PDT, where photosensitizers are distributed at the subcellular level [59].

The second effect of PDT is on the vasculature. A proper vascularization/angiogenesis is a key process in cancer development [60], the importance of which is highlighted by the appearance of necrotic and low-oxygen regions inside tumors in the presence of an unorganized formation of blood vessels. Damaging the existing vasculature or inhibiting the formation of new vessels thus has detrimental consequences for tumor proliferation [61]. Damaging the tumor vasculature has been shown to be an important factor in PDT efficacy. For example, much of the therapeutic effect of hematoporphyrin derivative appears to be largely due to the consequences of disrupted blood flow [62]. Following PDT, for example, endothelial cells round up, widening the inter-endothelial cell junctions and exposing the underlying tissue. Moreover, the damaged endothelial cells may release clotting factors, activating platelets [63]. The activated platelets interact with the exposed sub-endothelium, leading to platelet aggregation, thrombus formation, and vessel occlusion [62,64] and inducing vasoconstriction, further decreasing blood flow [65]. The impaired blood flow and blood-vessel destruction will therefore result in tissue hypoxia, nutrient deprivation, and tumor destruction [66].

The third mechanism of PDT-induced tumor destruction is the initiation of an inflammatory response that is followed by host tumor immunity. PDT-induced oxidative stress can upregulate the expression of heat-shock proteins, transcription factors related to inflammation, and the release of inflammatory cytokines [67]. Moreover, tumor cell death by PDT is accompanied by the release of proteins and other molecules, called damage-associated molecular patterns, which can elicit a strong inflammatory response. Following the immediate inflammatory response of the innate immune system, host anti-tumor immunity can develop. Anti-tumor immunity was first discovered when lymph node cells from PDT-treated mice were transplanted to naive hosts and induced the suppression of subsequent tumor challenges [68]. Even though the adaptive immune reaction is not essentially important in the initial tumor damage, it primes the host for recurrences of similar tumors by the formation of tumor-specific memory cells [69]. The acute inflammatory response following PDT, resulting in the phagocytosis of apoptotic and necrotic tumor cells, forms the basis for this mechanism.

Since its regulatory approval over 30 years ago, PDT has been the subject of numerous studies and has proven to be an effective form of cancer therapy. For example, Kaposi’s sarcoma (KS) is an angioproliferative neoplasm of endothelial origin, associated with human herpes virus 8 and human immunodeficiency virus (HIV), that has been effectively treated with PDT [70]. Several protocols have been developed, and complete remission with excellent cosmetic results were observed [71,72]. Other clinical trials and preclinical studies are ongoing; however, despite the clinical success reported, PDT is still underexploited in the clinic. This is also mainly due to the fact that directly activated light-driven therapies are limited to applications on surface tissues, i.e., shallow-lying tumors and in the internal part of surgical cuts [73]. The concept PDT-enhanced radiotherapy (X-PDT) has been developed to exactly bypass the limitation of the impossible activation of efficient PDT in deep tissues by exploiting the energy of ionizing radiation already employed in the diagnosis and treatment of oncological pathologies. To overcome this issue, many efforts have been devoted to developing near-infrared absorbing photosensitizers; however, near-infrared light can travel less than 2 cm into the human body and thus does not allow the treatment of deep tissue malignancies.

## 4. Principles of Radiotherapy Enhanced by Photodynamic Therapy (X-PDT)

**Figure 3:**
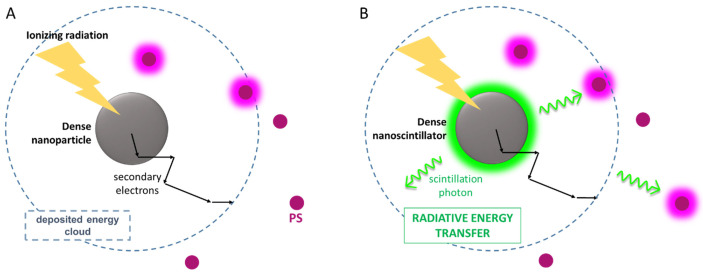
(**A**) Scheme of the radiosensitization effect. Upon irradiation, the dose deposited in the environment around a nanoparticle is localized in tumor tissue in a given volume, which is determined by the total diffusion length of randomly scattering secondary electrons (black arrows) generated upon interaction of the ionizing radiation with the dense material. This enables the excitation of the singlet oxygen photosensitizers (PS) included in the diffusion, whose PDT activity is added to the generation of ROS species in the aqueous biological exploited in radiotherapy. (**B**) Scheme of active radiosensitization exploiting a radiative energy transfer mechanism. By using a luminescent nanoscintillator, the photosensitizers can also be activated by absorbing the photons emitted by the dense nanoparticle or by recovering part of the energy deposited inside the nanoparticle.

As discussed above, the energy of X-rays used in clinical RT ranges from hundreds of keV to MeV. Consequently, most of the best photosensitizers that can be used for cancer PDT cannot be activated in these conditions because their low density does not enable efficient interaction with these highly energetic photons. The basic concept of the X-PDT approach is to exploit a physical transducer to harvest the X-ray energy and, consequently, to trigger the photosensitizers to produce the cytotoxic species required for the destruction of the cancer cells [74]. As illustrated in Figure 3A, the first idea of the X-PDT model relies on the use of scintillating dense and high-atomic-number (Z) nanoparticles (for instance, metal-containing nanoparticles), able to efficiently stop the ionizing X-ray radiation, according to the photoelectric effect cross-section that varies as (Z/E)^n^, with n ≅ 3–4 [75]. Thus, dense materials can improve the interaction with the ionizing radiation in RT by enhancing the deposition of energy in the proximity of the malignant cells. After interaction, the ionizing radiation produces charge carriers that can directly enhance the ROS production in the neighborhoods of the ionizing event (Section 2) because of the incremented amount of deposited energy (Figure 3A). In the case that a nanoscintillator is found to be in proximity with PDT agents, the additional secondary charges produced can interact directly with the photosensitizers encountered during their diffusion, thus activating their cytotoxicity. Notably, this effect always occurs in the presence of materials significantly denser than the biological aqueous environment, independently from their luminescence properties, taking the name of radiosensitization effect (*vide infra*, Section 4.1).

In the case of resonance between the nanoscintillator emission and photosensitizer absorption spectra, the fluorescence from nanoparticles can be absorbed by the photosensitizers, thus further enhancing the generation of additional ROS by an active radiosensitization, which is the core of the first X-PDT concept (Figure 3B, Section 4.2).

Inorganic nanomaterials such as metal oxides (HfO_2_ or ZnO) and lanthanide oxides (Ln_2_O_3_) as well as fluorides and silica-based nanostructures have been proposed for X-PDT application. Indeed, they can present intrinsic and extrinsic dopant emissions in the blue–green spectral range, suitable to excite organic conjugated photosensitizers such as fluorescent porphyrins [19,27,76,77,78,79]. Additionally, metal–organic frameworks containing a heavy metal, such as Zr or Hf, coupled to photosensitizers have shown attractive scintillating performances for therapeutic applications [78,80]. Another approach to perform PDT is based on the use of injected medical radioisotopes that are able to produce Cherenkov radiation suitable to excite the most common photosensitizers in the neighborhood of the malignancy, enabling a therapy with high selectivity [8,81,82]. However, the Cherenkov-radiation-based PDT efficacy is significantly limited due to the low number of photons generated by radionuclides with respect to classical RT, given that the photon fluence from radionuclides is in the order of nJ cm^−2^ while the fluence from external beams radiation is about mJ cm^−2^ [83,84].

The X-PDT modality therefore enables overcoming the intrinsic limit of the shallow activation of PDT in tissues. This breakthrough result triggered many studies, which demonstrate the more efficient tumor-cell killing with X-PDT compared with RT alone. This finding demonstrated that the X-PDT must not be considered as a trivial PDT or RT derivative but is essentially a PDT and RT synergistic combination [85]. Nevertheless, in order to assess the use of X-PDT as a clinical practice, several challenges have to be overcome. The first one is, obviously, to realize efficient but minimally toxic and safe nanoscintillators to be used in humans. The requirement of non-toxicity of the drug is of course mandatory to avoid additional stress to the organism and therefore justify the use of these materials, only taking their beneficial effects on the enhancement of the therapy efficacy. This point has been partially achieved since several examples of efficient X-PDT agents have been demonstrated as non-toxic (Table 1).

The second challenge is to ensure the targeted delivery to sick tissues. A large family of nanomaterials has been investigated for biomedical applications due to their biocompatibility and low toxicity, as well as the tunability of their structural and electronic properties. In parallel, the research focused on this has been developed to identify modification and functionalization strategies for more precise control of drug loading, delivery, or specific targeting of nanoparticles for drug delivery, as well as diagnostic and therapeutic applications [93,94]. This is a critical point for the development of any kind of drug or contrast agent, which usually limits it effective use. In Section 5, we discuss some of the selected strategies to provide the X-PDT agents the ability to selectively target cancer tissues. The third ongoing X-PDT challenge, discussed in detail below, is the modeling of the mechanism promoting its the effective therapeutic enhancement. Its relationship with the involved cytotoxic pathways is not yet fully understood, but it may deal with the complex physics involving energy sharing at the nanoscale between the nanoscintillators and the PDT agent. In this framework, the description of the energy-sharing mechanisms cannot be fully satisfied only by the picture of the very first X-PDT shown in Figure 3. A complete description is still lacking; thus, in the following paragraphs, we present diverse architectures of X-PDT systems with the explanation of the expected scintillation phenomena triggering the therapeutic outcomes and a discussion of their potential as X-PDT agents.

### 4.1. The Physics of X-PDT: The Radiosensitization Effect

The optimization of the energy sharing between the energy transducer and photosensitizers is the key point to develop efficient X-PDT systems. The PDT agent activation can indeed follow different pathways exploiting the passive radiosensitization effect or by direct active energy transfer from the nanoscintillator. As introduced above, the term ‘radiosensitization’ indicates the effect of increasing the localized deposited dose due the presence of dense nanoparticles in the biological water-based medium, for example, in the cellular cytoplasm. This is, for instance, what happens if we inject gold nanoparticles (AuNPs) in the tissues [95]. AuNPs’ therapeutic actions rely on the increment of the fraction of the X-rays energy deposited close to the particles themselves, which has been proven to induce additional hydroxyl radical creation with the increment of the DNA strands damaged up to 150%. 

To better understand the radiosensitization mechanism, we should recall that we have a limit to the amount of material that we can safely inject into organisms. For nanoparticles, these amounts are typically up to few μg each milliliter [85]. Thus, taking into account a material density between 3 and 9 g cm^−3^, the maximum calculated concentration of spherical particles with a reasonable radius of 50 nm that can be injected is around 10^−4^ μM. This means that water is still the most abundant material in any tumor tissue, and consequently, the primary absorber of the incident X-ray radiation, since the probability that the primary event occurs in the nanoparticles is extremely low. Upon primary interaction with water, the X-ray photons are converted in the swarm of secondary high-energy diffusing charges that move within the medium, depositing their energy through a series of random inelastic scattering events. During their diffusion, the charges can lose their energy by directly triggering the formation of the ROS species or activating the luminescence when encountering a dense nanoscintillator. Therefore, the indirect energy sharing through secondary events is the principal mechanism for the activation of the luminescence of nanoscintillator suspensions. Even in the case of a direct primary event, simulations demonstrate that with gadolinium oxide nanoparticles with a diameter of 10 nm, only a very small percentage of the deposited X-ray energy is stored in the nanoparticle, while most of the energy is transferred to the medium outside the nanoscintillator to distances of up to one hundred nanometers [96]. The effective amount of incorporated energy depends on the energy of the ionizing radiation and on the nanoparticle size and composition, which sets the material’s stopping power. Thus, a satisfactory X-PDT system should be designed to incorporate as much of the available deposited energy as possible to activate the PDT effect efficiently and promptly, but with not too much focus on the optimization of the possible primary interaction.

### 4.2. The Physics of X-PDT: Energy Transfer Mechanisms for PDT Activation

Another pathway to sensitize the PDT agents is by the direct recombination of diffusing secondary charges generated around the nanoparticle on the PDT moiety (Figure 3A). For this process, neither matching the nanoparticles and photosensitizers electronic properties nor the physical connection between the dense particle and the photosensitizer is required. Nevertheless, the two objects should be placed at a reasonably short distance in the biological environment, a distance comparable to the diffusion length of moving charges. The drawback of this mechanism, which basically occurs in any mixture of nanoscintillators and photosensitizers, is indeed that its yield is set by the statistical probability that a photosensitizer crosses the path of a relaxing traveling charge to become its natural recombination center, which depends on the concentration and diffusivity of photosensitizers [97]. In addition, from a practical point of view, the manipulation, delivery, and localization of mixtures containing uncoupled materials is quite a difficult task.

An alternative energy-sharing pathway that does not strictly depend on relative distances is the radiative energy transfer process [98], which implies the emission of a photon by scintillation from the dense particle and its subsequent absorption by a PDT agent (Figure 3B). In this case, the nanoscintillator emission energy must match the absorption spectrum of the selected photosensitizer. The efficiency of radiative energy transfer strictly depends on the scintillation quantum efficiency, i.e., the light yield (LY), which is usually defined as the number of photons emitted with respect to the fraction of deposited energy and expressed in ph MeV^−1^. The LY value depends on several parameters including the type and energy of the ionizing radiation and the photoluminescence quantum yield of the nanoparticle, which can be as large as an increase near to 100% in several types of scintillating nanomaterials [99,100,101]. Lastly, the efficiency of the radiative ET is dependent on the concentration of the absorbing moiety, i.e., the PDT agent. For these materials, the maximum concentration within the biocompatibility limits is usually around 10 μg mL^−1^, [102] thus the effective absorption of scintillating photons emitted isotropically by the nanoparticles is quite low. The latter limiting factor makes the radiative energy transfer a poor sensitization pathway to produce ROS in X-PDT: even in the ideal case where the process shows a total reabsorption of scintillation photons, the number of activated PDT agents will be low, hindering a significant cytotoxic effect on cancer cells.

This issue can be partially addressed by exploiting a non-radiative energy transfer mechanism between the nanoscintillator and the photosensitizer. Here, the energy is transferred from an energy donor system to an energy acceptor without the production of electromagnetic radiation. In general, the non-radiative energy transfer can be distinguished in Förster-type or Dexter-type mechanism, depending on the physical mechanism that rules the interaction in the donor/acceptor pair. The Förster mechanism operates by an electrostatic coupling of the transition dipole moments of the donor/acceptor pair. It can be represented as a simultaneous non-radiative de-excitation in the energy donor and excitation of the acceptor. Its rate falls off with the inverse sixth power of their distance, depends on their mutual orientation, and requires an energetic matching of the two transitions involved [103]. Quantitatively, this match is expressed by the overlap integral of the emission spectrum of the donor and the absorption spectrum of the acceptor. The Förster transfer rate can be calculated considering the donor emission lifetime; thus, given the usual fluorescence lifetimes for excited singlet states, the Förster mechanism is effective at distances of up to several nanometers [104]. Importantly, since the Förster transfer rate depends on the transition moments of the involved electronic transitions and the singlet–triplet transition moments being very small as they are dipole prohibited, the triplet–triplet Förster energy transfer is usually very slow. In general, this may be compensated by the possibly of a very long lifetime of the donor triplet state of the donor. On the other side, the Dexter or exchange mechanism is a short-range interaction that requires the physical overlap of the wave functions of the two chromophores’ electronic states; it is effective at a distance of around one nanometer. It can be represented as a simultaneous transfer of an excited electron from one partner to the other and of its unexcited original counterpart in the opposite flow. Considering the interaction involved, the Dexter transfer is therefore effective for intermolecular distances below one nanometer. This means that if the components are effectively in contact, Dexter transfer can be competitive with the Förster mechanism for singlet excitation energy transfer and vastly faster than the Förster mechanism for triplet excitation energy transfer.

**Figure 4:**
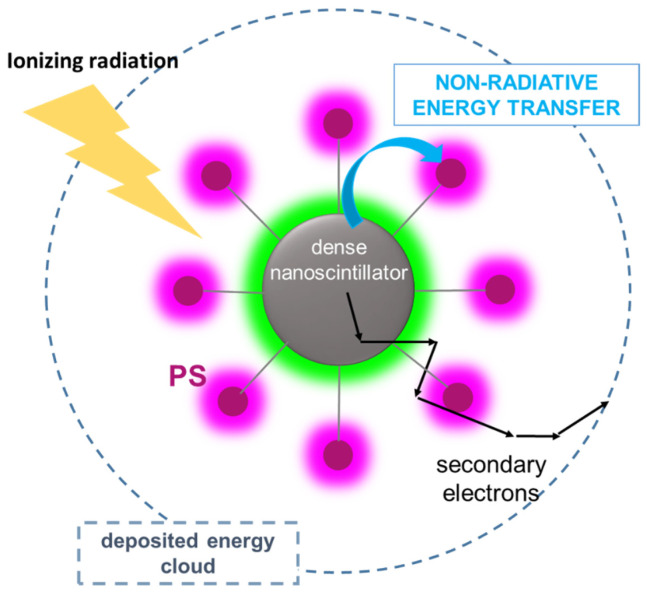
Scheme of active radiosensitization exploiting non-radiative energy transfer mechanisms. By anchoring the photosensitizers (PS) to the surface of a luminescent nanoscintillator at short distances (<10 nm), the energy stored in the dense core upon interaction with ionizing radiation can be exploited to activate the PS by fast non-radiative energy transfer without the need of the generation and reabsorption of a photon. If the non-radiative transfer is not complete, the residual emission for the nanoscintillator can also be reabsorbed by PS molecules, achieving an additional radiative energy transfer channel as showed in Figure 3B.

As shown in Figure 4, to exploit the non-radiative energy transfer, the photosensitizer must be placed in close proximity to the nanoscintillator, in a multicomponent stable architecture based on chemical or physical bonding. In such a configuration, the energy deposited in the dense material can be fully harvested by photosensitizers by means of both radiative and non-radiative energy transfer and subsequently exploited to create the singlet oxygen species by a second non-radiative energy-transfer step [10]. One potential architecture of X-PDT systems exploiting the non-radiative energy transfer [78] is represented by the metal–organic frameworks (MOFs) containing heavy metal elements, i.e., the high energy radiation interacting in dense nodes, linked together through photosensitizer molecules. In this architecture, the proximity of nanoscintillators and ligands allows an efficient non-radiative energy transfer and the prompt activation of the ligands and the therapeutic effects. Recently [105], diverse Hf-based nanoscale metal–organic frameworks (<100 nm in sizes), namely Hf_6_-DBA and Hf_12_-DBA (DBA = 2,5-di(p-benzoato)aniline), showed an energy-transfer-mediated enhancement of hydroxyl radical generation of 33.6% or 55.3%, compared with water, with an X-ray dose of up to 10 Gy. 

Similar results have been obtained with other hybrid nanosystems of biocompatible chrysotile for RT implementation. The results revealed that a tailored surface functionalization, including an *ad hoc* spatial arrangement of photosensitizer molecules, allows the boosting of the nanosystem performances, demonstrating how the non-radiative transfer from the nanoscintillator can be an efficient pathway to activate the photosensitizer moiety [85]. According to what was discussed above, in the following paragraph, we depict a guideline for the conception of efficient X-PDT systems, based on the scenario outlined in Figure 4. In this case, a duality of effects can occur, namely radiosensitization and non-radiative energy transfer from the nanoscintillators to the photosensitizers; thus, this configuration would enable the X-PDT system performances and the related curative outcomes to be enhanced.

### 4.3. X-PDT Agents: Design and Optimization

The understanding and engineering of the physical mechanism occurring in the X-PDT systems (radiosensitization and non-radiative energy transfer processes) are crucial for the design and development of optimized X-PDT agents for the enhancement of RT’s therapeutic effect. Hereby, we propose an affordable and feasible guideline for the manufacture of cutting-edge X-PDT systems, based on considerations from the point of view of the physical phenomena occurring within the nanosystems. Notably, the following considerations are valid for both the cases in which the nanoscintillators are activated by an external source of ionizing radiation [85] or if radionuclides are embedded in the X-PDT system architecture for spontaneous activation [106].

According to the working principle of non-radiative energy transfer mechanisms, the electronic properties of the nanoscintillator must be engineered to have a photoluminescence yield as close as possible to 100% and to maximize the resonance between its luminescence spectrum and the absorption of the selected PDT moiety [67]. In such a way, we avoid parasitic energy losses when scintillation charges recombine in the nanoparticles activating its emissive states, ready to transfer the stored energy to the PDT systems. As shown in Figure 4, when the PDT moiety is anchored on the nanoparticle surfaces, their distance is short enough to make both Dexter and Forster mechanisms active and competitive and, more importantly, to enable a transfer with a rate above the gigahertz frequency. Since more than one energy acceptor is present on the surface, the global transfer rate, which increases with the number of available energy acceptors, can be fast enough to guarantee a 100% energy harvesting, even when the nanoscintillator luminescence is partially quenched by competitive mechanisms. [107] This condition can therefore relax the request to have a nanoscintillator with maximized photoluminescence yield, which is a difficult task especially when using metal oxide nanoparticles or semiconductor nanocrystals emitting in the blue region where most of photosensitizers absorb [79]. At this point, if the arrangement of PDT agents can be properly controlled to avoid detrimental intermolecular interactions on the nanoscintillator surfaces, i.e., undesired aggregation phenomena that can affect the PDT system ability as singlet oxygen sensitizers, the non-radiative energy transfer can be properly exploited to recover the energy stored in the dense core of the X-PDT system.

**Figure 5:**
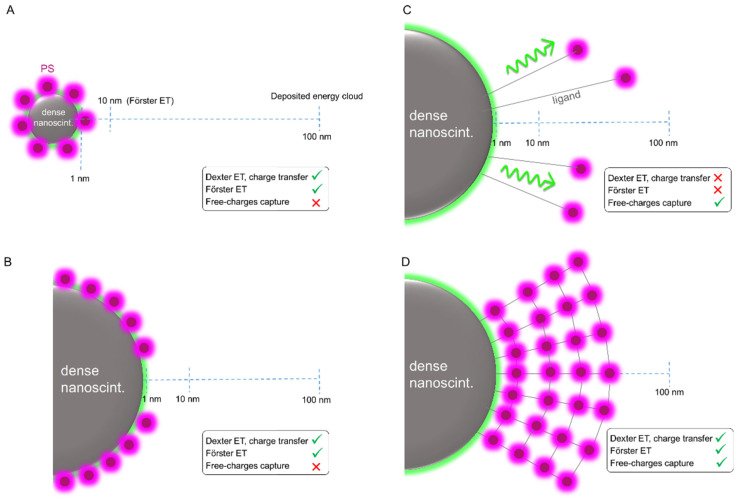
Examples of X-PDT system architectures. Dashed lines mark the reference length values of the mechanisms involved in the energy sharing between the nanoscintillator and the PDT photosensitizers (PS). (**A**) A small nanoscintillator with a size below 10 nm is decorated by PS anchored to its surfaces, thus enabling both Dexter and Forster non-radiative energy-transfer processes. (**B**) A nanoscintillators of size 50 nm can retain more energy during the scintillation process and host a number of PS molecules large enough to maximize the non-radiative transfer rate. (**C**) The PS molecules are physically bonded to the large nanoscintillator at a distance larger than the typical interaction radii of non-radiative energy transfers to harvest the energy deposited outside the nanoparticle by the diffusing secondary electrons. (**D**) The PS molecules and the nanoscintillator are arranged in a core/shell architecture to exploit the non-radiative transfer and to efficiently harvest the energy deposited outside the dense core as diffusing free charges.

However, we should recall the imbalance between nanometric sizes, and the dimensions of the process involved in the scintillation mechanism, such as the mean distances of the cascade phenomena involved (e.g., free charge thermalization length) discussed above. Figure 5A shows a sketch of the length scales involved in the scintillation process of the X-PDT system based on a 10 nm dense scintillating core obtained by detailed simulations of the process [96]. In such a configuration, it is easy to conclude that most of the ionizing radiation energy is deposited outside the dense particle upon primary or secondary interaction, thus making standard radiosensitization the only effective pathway to active the photosensitizers or other nanoscintillators in the aqueous environment. A potentially more convenient design is therefore the one shown in the simplified sketch of Figure 5B. In this case, the X-PDT system core is made by a dense nanoscintillator with a size of up to 100 nm, with the surface covered by the PDT moiety. This configuration has the double advantage of (i) enhancing the probability of a primary interaction and being able to store more energy thanks to its larger volume and (ii) triggering the activation by non-radiative energy transfer of a much larger number of photosensitizers attached to the surfaces of the nanoparticle. Accurate modelling of the radiation matter interaction at the nanoscale is crucial to point out the ideal size of the X-PDT core, which depends on the type and density of the material employed and the maximization of the PDT sensitization by respecting the constraints imposed by biocompatibility and pharmacokinetics.

It is worth noting that there is not yet any univocal proof that the activation of PDT by non-radiative energy transfer is the best strategy to maximize the radiosensitization and therapeutic effects. Current research needs to carefully investigate this point, because otherwise, the X-PDT architecture can be further simplified. If the non-radiative ET can be neglected as a sensitization channel, the PDT moiety can simply be connected to the nanoscintillator in order to be confined in a volume corresponding to the region in which we have the maximum energy deposition probability upon secondary events and charge recombination, as can happen in composite bulk scintillators [108]. Ideally, this can be achieved by exploiting long binding ligand chains, as showed in Figure 5C. In such a way, the secondary event energy cascade will promote a localized water hydrolysis and the sensitization of the PDT agents, while at the same time avoiding synthetic problems related to controlled surface functionalization with large amounts of photosensitizers. Interestingly, this configuration will make the nanoscintillator luminescence accessible again, which can be further exploited for therapeutic purposes, i.e., by using UV-emitting nanoparticles to directly damage the cellular DNA [109]. Nevertheless, the discussion above lead to the conclusion that a potentially ideal configuration for the best X-PDT material would be the one showed in Figure 5D. Here the nanoscintillator and photosensitizers are organized in a core-shell architecture, with a photosensitizers-based shell which size and structure are designed to maximize the energy harvesting by simultaneous capture of free charges and by non-radiative energy transfer around the dense core.

## 5. Targeting and Surface Functionalization of X-PDT Systems

Inorganic, organic, and hybrid nanoparticles and nanoscintillators are being considered for a plethora of biomedical applications due to their biocompatibility and low toxicity as well as the tunability of their size, porosity, crystallinity, and shape. Therefore, extensive research has been developed to identify the surface modification and functionalization strategies for a more precise control of drug loading, particle dispersion, blood circulation, accumulation, and specific targeting of nanoparticles for drug delivery and diagnostic and therapeutic applications [110,111]. 

Specifically, tumors are characterized by structural and molecular peculiarities that differ from those of the healthy tissues. Thus, this diversity is exploited to develop targeting strategies of nanoparticles to be used for several oncological therapies with a minimal impact on healthy tissues and reduced side effects: due to the structural features of the tumor vasculature, involving chaotic growth, a disordered structure, irregular shape and diameter, and poor lymphatic drainage, nanometric particles can penetration through the blood–tissue barrier and accumulate in the tumor tissue [93] via the so-called “enhanced permeation and retention” (EPR) effect. In this frame, the characteristics of the X-PDT agents also need to be optimized to enhance the EPR effect and maximize the tumor accumulation rate [112]. In any X-PDT nanosystem, therefore, the surface charge and chemistry qualities together with the dimensional/morphological properties play a crucial role in ruling its water solubility, selectivity, stability, and thus, the circulation time in order to avoid rapid elimination by the phagocyte system. For instance, the X-PDT system’s surface should be made hydrophilic through grafting by water-soluble polymers (generally PEGs) or through the creation of silica core–shell nanoparticles that can embed various photosensitizers [86]. However, in general, this kind of passive targeting of nanoparticles is not selective for tumor cells, and it must be integrated with the addition of active targeting motifs for specific accumulation in tumors. For instance, tumor cells express peculiar receptors; thus, the functionalization of X-PDT nanomaterials with receptor-targeted complementary ligand moieties enables the localization of the particles and therefore the therapeutic effects specifically into cancer cells, while improving their accumulation and time of retention [113]. This active targeting strategy implies covalent or non-covalent binding of the X-PDT agent to specific biomolecules, i.e., low-molecular-weight receptor-binding molecules such as peptides and vitamins, as ligands of therapeutic nanomaterials that are capable of selective interaction with the corresponding molecules on the surface of cancer cells. Table 2 reports some examples of nanomaterials that have been properly functionalized to show an active targeting. For example, patients’ cancer cells often express a high level of folate receptor due to their metabolic needs. Folic acid is indeed a validated method to direct nanoparticles to cancer cells [114]. It has been observed that the functionalization with folic acid of mesoporous silica-coated nanoparticles labelled with the protoporphyrin IX and of polymeric nanoparticles incorporating verteporfin as a photosensitizer increases the affinity and uptake in cancer cells via the endocytosis pathway mediated by folic acid receptors [12,86], as recently shown with gold nanoclusters functionalized with mAb trastuzumab and/or folic acid as a single and dual targeted radiosensitizers for the enhancement of radiation therapy efficacy of human breast cancer [115]. A more specific delivery strategy exploits monoclonal antibodies and their recombinant derivatives that are target-recognizing molecules commonly used in drug delivery [116]. Monoclonal antibodies are synthetic immune-system proteins capable of binding to the surface of a variety of cancer cells, avoiding normal cells. For instance, an X-PDT nanosystem based on nano-graphene oxide and integrated by integrin αvβ3 monoclonal antibodies has been validated to improve the photosensitizer cancer-killing efficiency with low side effects [117]. Another case of successful targeted surface coating and functionalization strategies is reported in [118], where the photosensitizer meso tetraphenylporphine (TPP) was solubilized using polymeric micelles prepared from polyethylene glycol phosphatidyl ethanolamine conjugate (PEG PE). TPP-loaded PEG PE micelles have been additionally modified with tumor-specific monoclonal 2C5 antibodies, resulting in significantly improved in vivo anticancer effects under the PDT conditions against murine lung carcinoma. Moreover, several peptides and other biopolymers have the advantages of increased tissue permeability, rapid internalization capacity, effective receptor binding, rapid clearance, and very mild antigenicity; these factors make them ideal for the targeted delivery of photosensitizers through a receptor-mediated targeting approach [119,120,121,122]. iRGD peptide has been proven to affect the uptake of iron oxide of superparamagnetic pancreatic cancer cells in vitro [123], and ultrasmall silica particles functionalized with peptide ligands and radioiodine have been exploited to enhance tumor-selective accumulation in αvβ3 integrin-expressing melanoma xenografts in mice [124]. Therefore, such smart molecules are still systematically developed through affinity selection from combinatorial libraries displaying small molecules, short peptides, antibodies and antibody fragments, engineered protein domains, and nucleic acid aptamers to improve the control of nanoparticle delivery and localization.

## 6. Conclusions

In this paper, we summarized the physical and photophysical mechanisms that are in involved in RT and PDT mechanisms, which are currently under investigation to achieve a combined X-ray-enhanced PDT for the improved treatment of deep tissue cancer. A critical view of the physical aspect of the process is mandatory to identify the appropriate guidelines for the development of optimized multicomponent nanomaterials to be tested in clinics. The imbalance between the nanoparticle dimensions and the dimensions of phenomena involved in the scintillation mechanism indeed leads to fundamental differences in the scintillation mechanisms when compared to monolithic bulk materials. The nanometric dimensions of the dense particles that are exploited to locally maximize the radiosensitization localized in sick tissues are indeed too small to be able to store a significant fraction of the energy transported and dissipated by secondary charges created during scintillation. This suggests that a classical design based on nanoscintillators functionalized with PDT moieties may be not the best one to properly sensitize the PDT effect, even if the system architecture is optimized to maximize the energy transfer yield from the nanoscintillator to the PDT moiety. We propose a general alternative layout of the X-PDT system in which the PDT functionality is placed around the nanoscintillators at distances of up to 100 nm to realize a harvesting network for the energy deposited outside the nanoparticle. A detailed spectroscopic and in vitro investigation is therefore necessary to validate this material design strategy, which can pave the way to the realization of an efficient X-PDT system and that could help shed light on the peculiar interaction of the ionizing radiations in the biological environment at a nanoscale. It should not be forgotten that the proposed nanomaterials should also possess good biocompatibility properties and must be designed in order to show selective accumulation properties in tumor tissues to maximize the therapeutic effect. Indeed, it is worth noting that despite huge progress being made in this direction in the last decade, the targeted delivery of nanoparticles is still one of the most challenging problems for the nanotechnologies that are developed for the treatment of malignant tumors and other diseases, as well as for targeted imaging and diagnosis. Success in fabricating efficient X-PDT agents and developing efficient delivery strategies would represent a breakthrough in the treatment of oncological diseases. The availability of an effective RT coadjutant would revolutionize the method currently employed for the treatment of many cancers, since the X-PDT agents may in principle be implemented in any RT treatment, including advanced methods such the Gamma Knife technique, to improve both the specificity and efficacy while limiting therapeutic side effects. This latter point is crucial as it contributes to the improvement of the patient’s quality of life, thus reducing the detrimental social/economic consequences ascribed to the need for additional care and collateral costs of the disease.

## Figures and Tables

**Table 1:** Examples of non-toxic X-PDT multicomponent nanomaterials developed recently.

Nanoscintillator	PDT Agent	Targeting Strategy	Studies	Ref.
Ce(3+)-doped lanthanum(III) fluoride nanoparticles	protoporphyrin IX (PpIX)	poly(D,L-lactide-co-glycolide (PLGA) microspheres	in vitro	[76]
mesoporous silica (passive X-PDT)	Rose Bengal (RB)	-	in vitro in vivo	[27]
chrysotile nanotube	Erythrosine B, Rose Bengal, meso-tetra(4-sulfonatophenyl)porphyrin	mPEG2K-phosphate (PEO)	in vitro	[85]
Mesoporous silica coated NPs	protoporphyrin IX (PpIX)	folic acid (FA)	in vitro in vivo	[86]
ZnGa_2_O_4_:Cr	ZnPcS Phtalocyanine	-	in vitro in vivo	[87]
Hf-based Metal-organic Frameworks	TCPP (tetrakis (4-carboxyphenyl) porphyrin)	-	in vitro in vivo	[88]
Hf-based Metal-organic Frameworks	5,15-di(p-benzoato)porphyrin (H_2_DBP)	-	in vitro in vivo	[89]
SiC/SiO_x_ core/shell nanowires	tetracarboxyphenyl porphyrin	-	in vitro	[90]
LiGa_5_O_8_:Cr	2,3-naphthalocyanine	mesoporous silica shells conjugated with cetuximab	in vitro in vivo	[91]
LaF_3_:Tb	Rose Bengal (RB)	-	in vivo	[92]
SrAl_2_O_4_:Eu^2+^	merocyanine 540 (MC540)	coated with two layers of silica	in vitro in vivo	[15]
co-doped ZnS (ZnS:Ag,Co) NPs	protoporphyrin IX (PpIX)	-	in vitro	[93]
Au nanoparticles	verteporfin (VP)	SH-PEG or SH-PEG-NH_2_	in vitro	[94]
(PLGA) polymeric nanoparticles	verteporfin (VP)	folic acid (FA)	in vitro	[12]

**Table 2:** Example of active targeting strategies of the nanoparticles (NP) employed for radiation-based therapies or diagnostics.

Type of NPs	Active Targeting	Application	Tests	Ref.
Mesoporous silica-coated NPs functionalized with protoporphyrin IX (PpIX)	Folic acid (FA)	X-PDT	In vitro; in vivo.	[86]
(PLGA) polymeric NPs incorporating verteporfin (VP)	Folic acid (FA)	X-PDT	In vitro	[12]
Gold nanoclusters	trastuzumab (Herceptin^®^) and/or folic acid (FA)	dual-targeted RT	In vitro	[115]
graphene oxide (NGO) functionalized with pyropheophorbide-a (PPa) and polyethylene-glycol (PEG)	integrin α_v_β_3_ monoclonal antibody (mAb);	tumor mitochondria-targeted PDT	In vitro	[117]
meso-tetraphenylporphine (TPP) loaded polyethylene glycol-phosphatidyl ethanolamine (PEG-PE) micelles	monoclonal 2C5 antibody (mAb 2C5)	PDT	In vitro; in vivo.	[118]
gold nanorods	nuclear location signal peptides (GNRs)	photothermal therapy (PTT)	In vitro; in vivo	[120]
poly (N,N-dimethylacrylamide) (PDMA), and near-infrared (NIR) light absorbing agent (hCyR) NPs	TAT peptide	photoacoustic therapy (PA)	In vitro; in vivo	[122]
iron oxide NPs	iRGD peptide	Multimodal probe (diagnostic)	In vitro	[123]
Cy5 dye-encapsulating core-shell silica NPs	polyethylene glycol (PEG) chains; ανβ3 integrin–targeting cRGDY peptide ligands	Multimodal probe (diagnostic)	In vitro; in vivo	[124]

## Data Availability

Not applicable, no data are reported.
